# Queen Conch (*Strombus gigas*) Testis Regresses during the Reproductive Season at Nearshore Sites in the Florida Keys

**DOI:** 10.1371/journal.pone.0012737

**Published:** 2010-09-15

**Authors:** Daniel J. Spade, Robert J. Griffitt, Li Liu, Nancy J. Brown-Peterson, Kevin J. Kroll, April Feswick, Robert A. Glazer, David S. Barber, Nancy D. Denslow

**Affiliations:** 1 Department of Physiological Sciences and Center for Environmental and Human Toxicology, University of Florida, Gainesville, Florida, United States of America; 2 Interdisciplinary Center for Biotechnology Research, University of Florida, Gainesville, Florida, United States of America; 3 Department of Coastal Sciences, University of Southern Mississippi, Ocean Springs, Mississippi, United States of America; 4 Fish and Wildlife Research Institute, Florida Fish and Wildlife Conservation Commission, Marathon, Florida, United States of America; Temasek Life Sciences Laboratory, Singapore

## Abstract

**Background:**

Queen conch (*Strombus gigas*) reproduction is inhibited in nearshore areas of the Florida Keys, relative to the offshore environment where conchs reproduce successfully. Nearshore reproductive failure is possibly a result of exposure to environmental factors, including heavy metals, which are likely to accumulate close to shore. Metals such as Cu and Zn are detrimental to reproduction in many mollusks.

**Methodology/Principal Findings:**

Histology shows gonadal atrophy in nearshore conchs as compared to reproductively healthy offshore conchs. In order to determine molecular mechanisms leading to tissue changes and reproductive failure, a microarray was developed. A normalized cDNA library for queen conch was constructed and sequenced using the 454 Life Sciences GS-FLX pyrosequencer, producing 27,723 assembled contigs and 7,740 annotated transcript sequences. The resulting sequences were used to design the microarray. Microarray analysis of conch testis indicated differential regulation of 255 genes (p<0.01) in nearshore conch, relative to offshore. Changes in expression for three of four transcripts of interest were confirmed using real-time reverse transcription polymerase chain reaction. Gene Ontology enrichment analysis indicated changes in biological processes: respiratory chain (GO:0015992), spermatogenesis (GO:0007283), small GTPase-mediated signal transduction (GO:0007264), and others. Inductively coupled plasma-mass spectrometry analysis indicated that Zn and possibly Cu were elevated in some nearshore conch tissues.

**Conclusions/Significance:**

Congruence between testis histology and microarray data suggests that nearshore conch testes regress during the reproductive season, while offshore conch testes develop normally. Possible mechanisms underlying the testis regression observed in queen conch in the nearshore Florida Keys include a disruption of small GTPase (Ras)-mediated signaling in testis development. Additionally, elevated tissue levels of Cu (34.77 ng/mg in testis) and Zn (831.85 ng/mg in digestive gland, 83.96 ng/mg in testis) nearshore are similar to reported levels resulting in reproductive inhibition in other gastropods, indicating that these metals possibly contribute to NS conch reproductive failure.

## Introduction

Queen conch (*Strombus gigas*) is a species of significant ecological and economic importance throughout its range. For example, the estimated economic value of the annual conch fishery in the Bahamas is approximately $4.457 million, representing 9,800 seasonal jobs [1]. The queen conch is also a large benthic invertebrate associated with coral reef ecosystems, and therefore could serve as an indicator species for toxic effects contributing to the decline of the Florida coral reef ecosystem. As a result of the queen conch population decline in Florida, a complete moratorium on the Florida conch fishery was declared in 1986 [2], [3]. The queen conch was listed under the Convention on International Trade in Endangered Species' (CITES) Appendix II in 1992 [4]. However, recovery of adult conchs in spawning aggregations within the Florida Keys has been modest. In 2001, the number of adult conchs in offshore (OS) spawning aggregations was estimated at 27,000, up from a lowest observed estimate of 5,750 in 1992, according to transect data collected by the Florida Fish and Wildlife Conservation Commission [3]. It is believed that little or no reproduction occurs in nearshore (NS) aggregations, and that this might contribute to the slow recovery of the population [2], [3]. A study of conch reproduction found that NS conchs failed to develop adequate gonad tissue for reproduction, but that translocation of NS conchs to the OS environment resulted in development of normal gonad tissue and reproductive activity within three months [2]. However, the causes of reproductive failure of NS conchs remain unknown.

Human impacts on coastal marine ecosystems are ever-increasing, and threats include inputs of nutrients, organic contaminants, and metals, as well as changes in temperature, decreasing ocean pH, and deoxygenation [5]. While many of these factors can theoretically affect reproduction, one plausible cause for reproductive failure in a NS marine gastropod is heavy metal exposure. A number of gastropod studies have related heavy metal exposure, in particular exposure to Cu [6]-[8] and Zn [6], [9], [10], to reduced fecundity – reproductive output usually measured in terms of egg laying. Despite the link between exposure to Cu and Zn and decreased reproductive output in gastropods, past studies consider mostly female-mediated effects at the individual level. In the Florida Keys, both male and female reproductive development is inhibited NS [2], [3]. Given that heavy metals are known to inhibit gastropod egg laying, and that general and point sources for metal contamination exist close to shore in the Florida Keys [11]–[13], our general hypothesis is that heavy metals are likely to contribute to the reproductive failure observed NS. For the present study, our specific hypotheses were:

1. that testis transcriptional data would identify candidate gene expression pathways affected by NS environmental stressors, and
2. that tissue concentrations of heavy metals in NS conchs would exceed those of OS conchs.

For this study we developed and used a microarray to identify gene expression differences between the testes of NS and OS conchs. Gene expression data was anchored in histopathology to provide a more complete understanding of the dysfunction in testis development in NS conchs. Additionally, we used inductively coupled plasma-mass spectrometry to quantify nine metals, including Cu and Zn, in conch tissues and to determine whether their concentrations correlate with histological and gene expression evidence of NS reproductive dysfunction.

## Methods

### Field collections

**Figure 1:**
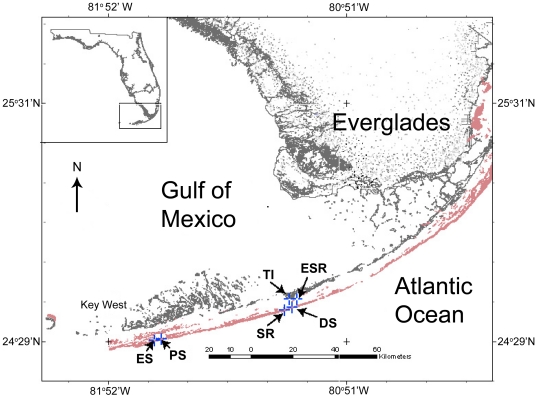
Nearshore (NS) and offshore (OS) queen conch sampling sites in the Florida Keys. Microarray construction: **ESR**, East Sister's Rock (NS); **ES**, Eastern Sambo (OS); **SR**, Sombrero Reef (OS). Microarray and real-time reverse-transcription polymerase chain-reaction experiments: **PS**, Pelican Shoal (OS); **TI**, Tingler Island (NS). Validation of 18S rRNA for real-time RT-PCR: **PS**; **TI**; **ES**; **ESR**; **DS**, Delta Shoal (OS). Inductively coupled plasma-mass spectrometry and histology: **PS**; **TI**; **ESR**, **ES**.

For this study, all conchs were collected in the Florida Keys (Figure 1). Tissue samples used for queen conch cDNA library construction were collected from Sombrero Reef on 9 June 2004 and from East Sister's Rock and Eastern Sambo on 15 March 2005. Tissue samples used for the comparison of reproductive (OS) versus non-reproductive (NS) queen conchs by histology, real-time reverse-transcription polymerase chain reaction (real-time RT-PCR), and metals analysis were collected from Pelican Shoal (OS) and Tingler Island (NS) on 15 February 2007 and from Eastern Sambo (OS) and East Sisters' Rock (NS) on 7 June–9 June 2007. Microarray analysis was conducted using 15 February 2007 samples from Pelican Shoal and Tingler Island. Sampling was conducted by free diving or SCUBA diving. Only adult queen conchs, identified by a fully flared lip [4], were collected.

In all cases, adult male conchs were collected live and transported immediately to the Florida Fish and Wildlife Conservation Commission's Fish and Wildlife Research Institute (FWRI) laboratory in Marathon, FL. Conchs were then euthanized and tissues were harvested. For molecular assays and determination of tissue metal burdens, gonad, digestive gland, neural ganglia, blood, and foot muscle samples were frozen immediately in liquid nitrogen. Frozen tissue samples were maintained at −80°C until further analysis. For histology, a piece of testis tissue approximately 1 cm^3^ in size from the middle of each conch's testis was placed in an individually labeled cassette and fixed in 10% neutral buffered formalin for a minimum of 7 days and retained for further processing.

### Histopathology

Histological examinations of the testes of experimental conchs were conducted as described in Delgado et al. [2]. Briefly, fixed tissue samples were rinsed in running tap water overnight, dehydrated in a graded series of ethanols, cleared, embedded in Paraplast (Leica Microsystems, Wetzlar, Germany), sectioned at 4 µm and stained with hematoxylin and eosin following standard histological techniques.

Testicular development was assessed histologically to determine reproductive capability of conchs. Conchs were classified into reproductive phases based on a maturity scale presented in Delgado et al. [2] (Table 1). In order to quantify the amount of testicular tissue present, a series of photomicrographs were taken to enable visualization of all gonadal tissue, resulting in 6–20 photographs for each specimen. Three photographs were randomly selected for each specimen, and the area of each testicular lobule in these photographs was determined using ImageJ software. A sperm scale based on the presence of developing sperm in the testis (spermatogonia  = 1, spermatocytes  = 2, spermatids  = 3, spermatozoa  = 4, vas deferens with spermatozoa  = 5) was recorded for each lobule. A spermatogenic index (SI) was calculated following methodology similar to that reported in Kofoed et al. [14]; the area of each lobule (mm^2^) was multiplied by the sperm scale for that lobule. A total SI was determined for each specimen by summing the SI for each lobule in the three photographic views.

**Table 1:** Reproductive phases used to describe testicular development in queen conch.

Reproductive Phase	Description
Early Developing	Only spermatogonia and spermatocytes present
Developing	All stages of spermatogenesis present; no spermatozoa in vas deferens
Spawning Capable	All stages of spermatogenesis present, spermatozoa present in vas deferens
Regressing	Empty lobules, resorption of spermatozoa. Some spermatogenesis occurring
Atretic	Lobules degenerating, resorption of spermatozoa, no active spermatogenesis
Regenerating	Only spermatogonia present
No Tissue	No spermatogenesis occurring and no spermatogonia present; this is an abnormal condition in adult males.

Modified from Delgado et al. [2].

### Construction, sequencing, and annotation of a normalized cDNA library

cDNA library construction was carried out following the method of Garcia-Reyero et al. [15]. Briefly, total RNA was isolated from 12 individual conch tissue samples (neural ganglia, gonad, and digestive gland from each of four conchs, one female and one male from NS and one female and one male from OS) using TRIzol (Invitrogen, Carlsbad, CA, USA) according to the manufacturer's protocol. RNA quality and integrity were assessed using the NanoDrop ND-1000 spectrophotometer (NanoDrop Technologies, Wilmington, DE, USA) and Agilent 2100 Bioanalyzer (Agilent Technologies, Santa Clara, CA, USA), respectively. 200 ng of each RNA sample was pooled and used as the template for the cDNA library construction and normalization using the SMART (Clontech/Takara Bio USA, Madison, WI, USA) and Trimmer (Evrogen Joint Stock Company, Moscow, Russia) kits, respectively. The normalized queen conch cDNA library was sequenced using a 454 GS-FLX pyrosequencer (Roche/454 Life Sciences, Branford, CT, USA) [16], at the University of Florida Interdisciplinary Center for Biotechnology Research (ICBR). Sequencing was conducted as described in Garcia-Reyero et al. [15]. Sequence assembly and annotation were performed as described in Farmerie et al. [17] using Newbler v1.1.03.24 and Paracel TranscriptAssembler. Minimum confidence for a match was set at e<1×10^−4^. Each sequence and its top 100 hits from BLAST search were retained in ICBR's BlastQuest database [17] and annotated with Gene Ontology (GO) information and with human and zebrafish (*Danio rerio*) homologues by referring to the NCBI Gene database. A queen conch oligonucleotide microarray was designed in an 8×15 k format consisting of 15,208 60-mer user probes and 536 internal control probes using the eArray service from Agilent. The microarray was submitted as a platform dataset to NCBI's Gene Expression Omnibus (GEO) database (GPL8934).

### Extraction of RNA from experimental samples

For microarray and cloning procedures, four biological replicate conch RNA samples were isolated from testis of male conchs collected in February, 2007, at Pelican Shoal (OS) and Tingler Island (NS) (Figure 1). One array from each group was determined to be of insufficient quality during the quality control process, and so the final sample size for microarrays was three individuals from each location, OS and NS. The OS individuals are considered to be the reference group, as they exhibit successful sexual reproduction, while NS conchs do not. Total RNA was extracted using the RNA STAT-60 reagent (Tel-Test, Friendswood, TX, USA), reconstituted in RNAsecure (Ambion), and DNase-treated with *Turbo* DNA-free (Ambion). In all cases, manufacturers' protocols were followed. RNA samples used for real-time RT-PCR assays were extracted using a more rigorous procedure, in which STAT-60 extraction of RNA was followed by gradient centrifugation through a 1.4 mL layer of 5.7 M CsCl, 0.5 M EDTA in the Beckman Optima-TLX ultracentrifuge and TLA120.2 rotor. RNA cleanup was performed using the Qiagen RNEasy Mini Kit. RNA quantity was assessed on the NanoDrop ND-1000 and quality was assessed using the Agilent 2100 Bioanalyzer. We have observed that un-degraded conch total RNA samples show no 28S rRNA band; therefore, no RIN can be calculated to quantify RNA integrity for these samples. This is likely due to the “hidden break” in invertebrate 28S rRNA that has been described by Ishikawa [18] and that is present in gastropods including *Haliotis rufescens* [19]. Despite the impossibility of calculating RIN, only high-quality samples for which Bioanalyzer profiles appeared to be un-degraded were used for further analysis.

### Microarray processing

RNA samples were labeled with Cy3-CTP and hybridized to the microarray following the Agilent Protocol titled One Color Microarray-Based Gene Expression Analysis v5.5 (publication no. G4140-90040). Specific activity of Cy3 label was at least 10.04 pmol/μL in each sample, and averaged 13.14 pmol/μL. Microarray scanning and feature extraction was performed at ICBR using an Agilent G2505B Microarray Scanner and Agilent Feature Extraction Software v9.5. All microarray data here reported are MIAME compliant; raw and normalized microarray data have been submitted to the GEO database (GSE17379), according to MIAME standards [20].

### Cloning of *S. gigas* 18S ribosomal RNA

3 μg of each conch RNA sample was reverse transcribed to produce cDNA using Invitrogen SuperScript II Reverse Transcriptase and random primers, per the manufacturer's protocol. 18S rRNA was cloned using primers designed in the program Primer3 [21] based on alignment of 18S rRNA from the gastropod *Bursa rana* (X94269.1) and the bivalve *Nucula sulcata* (AF207642.1) (Table 2). 18S rRNA primers were used in a PCR reaction with Invitrogen Taq polymerase, according to the manufacturer's protocol. PCR products were cloned in the pGEM-T Easy vector (Sigma-Aldrich, St. Louis, MO, USA) and Invitrogen One-shot Top10 chemically competent *E. coli* cells, per the manufacturer's protocols. The sequence of the cloned 18S rRNA fragment was confirmed by Sanger sequencing at ICBR (GU198749).

**Table 2:** Primers for 18S rRNA cloning and for real-time RT-PCR.

Sequence	Purpose	Direction	Primer Sequence (5′–3′)	Amplicon Size (bp)	Accession Number
18S rRNA	Cloning	Forward	GTT TCC CAT CCT ACG CTT CC	636	GU198749
		Reverse	AGA CAA ATC GCT CCA CCA AC		
18S rRNA	Real-time	Forward	TCG GTC TTA TTT TGC TGG TTT	226	GU198749
		Reverse	ATC GCT AGT TGG CAT CGT TT		
Ctr1c	Real-time	Forward	ACA AGG GCG GAA GAA GAA GT	158	UF_Sgi_AF_100593
		Reverse	GGC TTT CAG TAC CCA AAC GA		
TepII	Real-time	Forward	GTC ACG GCT GAC TCC TTC TC	151	UF_Sgi_AF_105314
		Reverse	TAA AGA ACA CGC CGA TCT CC		
GST	Real-time	Forward	TAT GGC AAG ACC AAC ATG GA	174	UF_Sgi_AF_101461
		Reverse	ATT CGC GTA AAA GCC AAA GA		
Stard7	Real-time	Forward	GCG CTG TTG CTG AAC ATA AA	183	UF_Sgi_AF_103703
		Reverse	CTT CTT GCA CAC CAT CTC GTT		

“Accession Number” refers to the accession for the sequence in NCBI (18S rRNA) or the UF ProbeName for the corresponding microarray probe. Transcripts: Ctr1c, copper transporter 1c; TepII, thiolester containing protein II; GST, Similar to Glutathione S-transferase; Stard7, StAR-related lipid transfer (START) domain containing 7.

### Real-Time RT-PCR

Copper transporter 1c (Ctr1c), thiolester-containing protein II (TepII), Similar to Glutathione S-transferase (GST), and Start domain-containing protein 7 (Stard7) were evaluated by real-time RT-PCR. Primers for transcripts of interest (Table 2) were developed from 454-derived cDNA library sequences using Primer3. All primer sets were verified using the same cloning and sequencing methods as in the section *Cloning of* S. gigas *18S ribosomal RNA*, above. The 638 bp clone of 18S rRNA was used to design a second set of primers that was more optimal for real-time RT-PCR. For all other clones, cloning primers were also used for real-time RT-PCR. Plasmids containing each cloned sequence were used to create a standard curve consisting of eight points in a serial dilution from 1×10^2^ through 1×10^9^ copies/reaction. Real-time RT-PCR was performed as a two step process. In step one, 2 μg DNase-treated RNA was reverse transcribed using Invitrogen's SuperScript II reverse transcriptase and random primers. In step two, real-time PCR reactions were run using SYBR Green Supermix (Bio-Rad, Hercules, CA, USA), per the manufacturer's protocol, on the Bio-Rad iCycler real-time PCR thermal cycler, using a two-step protocol with an initial denaturation at 95°C, followed by 40 cycles of denaturation at 95°C and annealing and extension at 58°C, during which real-time quantification was enabled. Following amplification, a dissociation curve was run beginning at 55°C and increasing to 95°C at 0.5°C intervals every 10s. Standards and experimental samples were run in duplicate, along with two negative controls for each gene: a “no reverse transcriptase (-RT)” control, in which DNAse-treated RNA samples were pooled and water was used in place of reverse transcriptase during the reverse transcription reaction, and a “no template control (NTC),” in which water was used in place of template cDNA during the real-time PCR reaction.

The use of 18S rRNA as a reference gene for RT-PCR was validated by measuring 18S rRNA by the method described above for 23 conch testis samples, collected in February, 2007, June, 2007, and March, 2009 (Figure 1). Initial quantity for each sample was calculated as 18S rRNA copy number/ng total RNA. Data were analyzed in JMP v8 using a two-way ANOVA on the factors “collection (date)” and “location (OS vs. NS).” This analysis showed nearly identical mean 18S levels between OS and NS, with no statistically significant difference according to ANOVA (Table S1). While no reference gene is perfect [22], [23], 18S rRNA appears to be the best internal reference for this experiment. 18S rRNA expression does vary across some sites and collection times, but is remarkably consistent for the samples here presented, collected in February, 2007. Moreover, at least one commonly used reference gene, β-actin, was differentially regulated between NS and OS in the microarray study (Table S2, ProbeName UF_Sgi_AF_101275), making it a poor candidate for our internal reference in real-time RT-PCR.

### Metal analysis by inductively coupled plasma-mass spectrometry (ICP-MS)

ICP-MS was used to determine levels of ^58^Ni, ^65^Cu, ^66^Zn, ^88^Sr, ^107^Ag,^ 111^Cd, ^118^Sn, ^202^Hg, and ^238^U in blood, digestive gland, foot, neural ganglia, and testis for male conchs collected in February and June 2007 field collections (n = 2-8, varying by sample type). Weighed tissue samples (approximately 50–200 mg) were acid digested to completion in 0.5 mL 67–70% optima grade HNO_3_ at 140° C for 2 hours. This was followed by addition of 0.5 mL 30% ultrapure H_2_O_2_ and further digested at 110°C until almost dry. The sample was quantitatively diluted to 5 mL using ultrapure water for a final concentration of 2% HNO_3_ and filtered through a 0.22 μm nylon syringe filter. The reconstituted samples were analyzed for total metal content using an XSeries 2 ICP-MS (Thermo Electron Corporation, Winsford, Cheshire, UK) with ^115^In as an internal standard. Samples were quantified against a seven point standard curve with standard concentrations 1, 5, 10, 50, 100, 500, and 1000 ppb each analyte. The lower limit of detectability for this assay was set at 0.5 ppb analyte in the digested sample.

### Statistical Analysis

Histological data were analyzed using JMP v8 (SAS, Cary, NC, USA). Differences in SI were analyzed using ANOVA with month nested within location. A student's t-test based on least square means of the nested ANOVA was calculated in JMP for all month and location combinations. SI was tested for homogeneity of variance (Levene's test) and normality of distribution (Kolmogorov-Smirnov test), and values were log_10_ transformed if assumptions were violated. Raw microarray data were imported into JMP Genomics v3.1 and analyzed as follows: Non-uniform spots were flagged and removed from the dataset. Next, 590 rows not containing at least two data points for each treatment group (OS and NS) were deleted, leaving 15,154 rows in the analysis. Array data were then median-centered prior to performing one-way ANOVA on the factor location (OS/NS) to identify differentially regulated transcripts (p<0.01 or p<0.05 for further analyses, FDR = 5%). Hierarchical clustering analysis of significant transcripts (p<0.01) was performed using the program Cluster [24] and visualized in the program Java TreeView [25]. Data were median-centered by gene and clustering was based on centered correlation and complete linkage. Real-time RT-PCR data were analyzed using the Kruskall-Wallis non-parametric test calculator available at http://elegans.wsmed.edu/~leon/stats/utest.html. ICP-MS data were imported into JMP v7 and analyzed for difference of means using two-way ANOVA, with the two factors being tissue and location; this analysis was followed by the *post hoc* Tukey-Kramer HSD test for multiple comparisons (p<0.05). For non-parametric correlation analysis, Spearman's ρ was calculated in JMP.

### Gene Ontology and Pathway Analysis

For microarray data, functional enrichment analysis of Gene Ontology terms was performed by Fisher's exact test using the FatiGO tool within the Babelomics suite [26]. All terms with a nominal p-value of p<0.05 (no *post hoc* correction) were considered to be enriched. Finally, Pathway Studio 7 (Ariadne Genomics, Rockville, MD, USA), operating on the ResNet 7.0 mammalian database updated with zebrafish annotation, was used to identify all shortest paths between genes falling under significantly enriched terms and cellular processes, in order to illustrate important connections within these biological processes, based on human and zebrafish (*Danio rerio*) homologs.

## Results

### Histopathology

**Figure 2:**
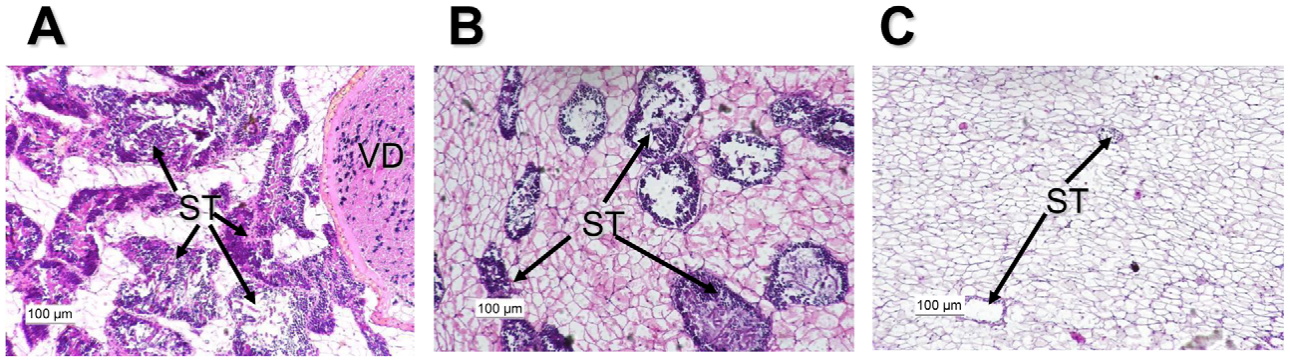
Histological sections of testis tissue from queen conchs captured in the Florida Keys in 2007. **A**. Testicular tissue from an offshore (OS), Spawning Capable male in February. All OS conchs captured in June had a similar appearance. **B**. Testicular tissue from a nearshore (NS), Developing male in February. **C**. Testicular tissue from a NS male in June showing little spermatogenic tissue or spermatogenesis. ST—spermatogenic tissue; VD—vas deferens.

Testis tissue from eight conchs (four OS, four NS) in February 2007 and seven conchs in June 2007 (two OS, five NS) was analyzed by histopathology (Table 3). All OS conchs from both months had normal, healthy testicular tissue present in >75% of each histological section (Figure 2A). In contrast, the amount of testicular tissue present in NS conchs was lower than OS conchs in both February and June (Figure 2B, C). Differences in gonadal development based on reproductive phase were not marked in February between NS and OS conchs. All conchs from both locations had testicular tissue undergoing active spermatogenesis in February, although a higher percentage of OS conchs (75%) were Spawning Capable compared to NS conchs (50%; Table 3). However, there was a significant difference in the SI between OS and NS conchs in February (t_11_ = 2.606, p = 0.024), with OS conchs having a SI value nearly 7 times greater than the NS value (Table 3).

**Table 3:** Summary of testicular development observed in queen conchs collected from offshore (OS) and nearshore (NS) sites in the Florida Keys in February and June 2007.

Gonadal Development	OS February (n = 4)	NS February (n = 4)	OS June (n = 2)	NS June (n = 5)
Early Developing	0	0	0	20
Developing	25	50	0	20
Spawning Capable	75	50	100	20
Regenerating	0	0	0	20
No gonadal tissue	0	0	0	20
SI	21.29±2.57^a^	3.74±0.72^b^	26.03±4.51^a^	0.60±0.47^c^

Data for reproductive phases presented as percentage. Data for spermatogenic index (SI) presented as mean ± SE. Superscripts indicate differences in SI (nested ANOVA, p<0.05).

In contrast, by June all OS conchs were Spawning Capable while only 60% of NS conchs exhibited active spermatogenesis, with only one NS individual Spawning Capable (Table 3). The SI was nearly 45 times higher in OS conchs compared to NS conchs in June (t_11_ = 6.05, p<0.001; Table 3). Overall, in both February and June the SI was significantly higher for OS conchs when compared to NS conchs. There was no difference in SI between months for OS conch (t_11_ = 0.245, p = 0.81), suggesting OS conchs were reproductively active from February through June. Interestingly, for NS conchs, the SI was significantly higher in February than in June (t_11_ = −4.49, p = 0.0009; Table 3) suggesting that the NS conchs were unable to maintain spermatogenic tissue during the reproductive season.

### Normalized cDNA library sequencing and assembly

Sequencing produced a total of 64,794,458 bases across 286,933 reads (average read length  = 225.8 bases) (Table 4). Sequences were submitted to the NCBI Sequence Read Archive (SRA) (experiment SRX017250). These sequences were assembled into 28,010 contigs, of which 7,740 matched to NCBI NR or NT database with e<1×10^−4^. Of 7,740 annotated sequences, at least one GO term was assigned to 3,971 (51.3 percent), human homologues to 2,688 (34.7 percent), and zebrafish homologues to 2,681 (34.6 percent). The microarray was designed with probes corresponding to all 7,740 annotated sequences and to 7,468 additional un-annotated sequences for a total of 15,208 user-defined elements.

**Table 4:** Information on conch transcriptome assembly.

Run	Bases	Reads	Average Length	Alias	SRA Accession
Titration	1,286,141	5,354	240.22	ERXZ1SM	SRR037030.3
Production-1	37,762,116	161,610	233.66	ES8A9FR	SRR037031.2
Production-2	15,882,529	67,779	234.33	ETMYUMG	SRR037032.2
Production-3	9,863,672	52,190	189.00	EU6CYYIF	SRR037033.2
Total	64,794,458	286,933	225.82	S. gigas 454	SRX017250.1

“Alias” refers to the run alias listed in the SRA database.

### Microarray analysis of testicular transcription

**Figure 3:**
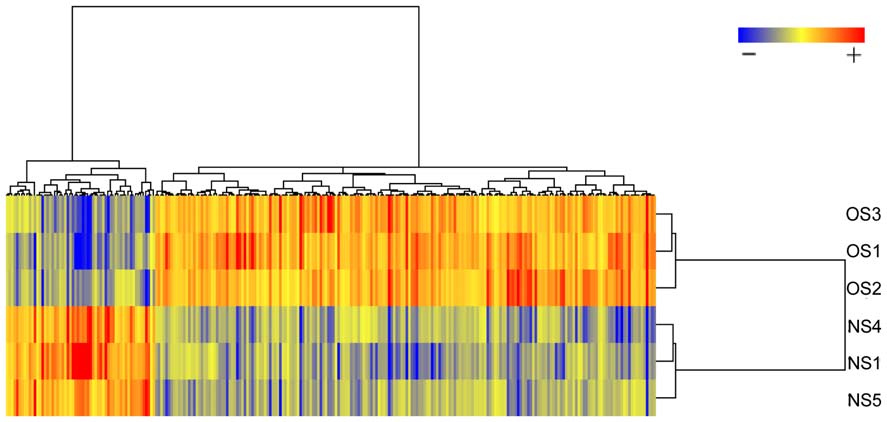
Hierarchical clustering of significantly differentially regulated genes in conch testis. Red color represents expression of a gene at a level greater than the row (gene) average, and blue color represents expression lower than the row average. The map shows a clear distinction between nearshore (NS) and offshore (OS) testis samples based on the 256 differentially-regulated transcripts. Approximately one-fourth of the regulated transcripts are up-regulated in NS relative to OS; the majority are down-regulated.

255 differentially-regulated probes (58 up and 197 down in NS with respect to OS conchs) were identified by ANOVA (n = 3, p<0.01, FDR = 5%) (Figure 3, Table S2). At a less stringent p-value, 1147 differentially-regulated probes (341 up and 806 down) were identified (p<0.05, FDR = 5%) (Table S2). Based on differentially-regulated probes, all OS and NS individuals clustered separately from one another, indicating that the identified set of transcripts show a clear difference between these two presumably outbred groups of wild conchs (Figure 3). Differentially regulated genes were predominantly down-regulated in this experiment; at a cutoff of p<0.01, the proportion of differentially regulated transcripts that are down-regulated was 77.4 percent. The two most up-regulated probes with annotation (p<0.01) were Similar to Glutathione S-transferase (GST, 15.24-fold up-regulated NS) and Collagen 1, Alpha 1 (COL1A1, 10.26-fold up-regulated NS). The two most down-regulated probes with annotation (p<0.01) were RIKEN CDNA F730014I05 Gene, a mouse genome sequence (13.83-fold down-regulated NS), and Dolichyl-phosphate Mannosyltransferase Polypeptide 2 Regulatory Subunit (Dpm2, 4.92-fold down-regulted NS).

**Figure 4:**
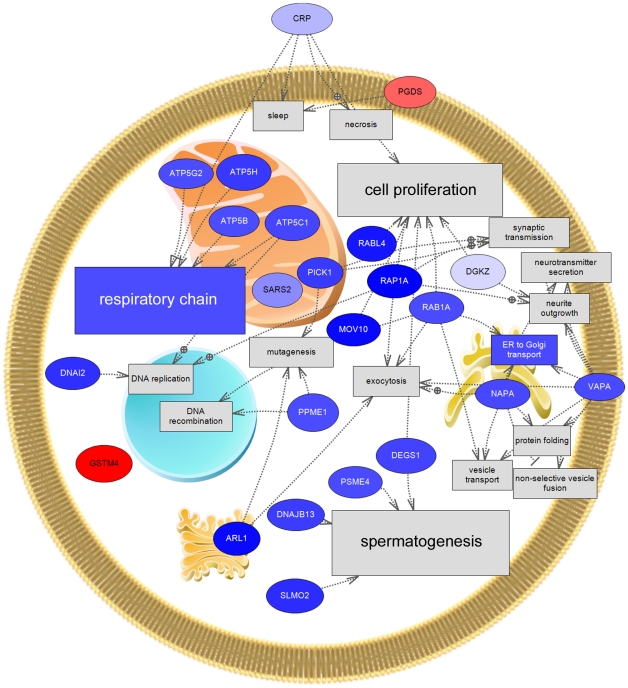
Pathway analysis of differentially regulated genes from conch testis microarray study. Pathway Studio (Ariadne Genomics) was used to find all shortest paths between human homologues of genes falling under significantly enriched GO Biological Processes in the testis. Red color represents up-regulation nearshore (NS); blue color represents down-regulation NS. Genes: ARL1, zgc:92883 (ADP-ribosylation factor-like 1); ATP5B, ATP synthase, H+ transporting, mitochondrial F1 complex, beta polypeptide; ATP5C1, ATP synthase, H+ transporting, mitochondrial F1 complex, gamma polypeptide 1; ATP5G2, ATP synthase, H+ transporting, mitochondrial F0 complex, subunit C2 (subunit 9); ATP5H, ATP synthase, H+ transporting, mitochondrial F0 complex, subunit d; CRP, si:ch211-234p6.13 (*Danio rerio* hypothetical protein); DEGS1, im:6909319 (degenerative spermatocyte homolog, lipid desaturase); DGKZ, hypothetical LOC571856 (similar to diacylglycerol kinase, iota); DNAI2, dynein, axonemal, intermediate chain 2; DNAJB13, DnaJ (Hsp40) related, subfamily B, member 13; GSTM4, glutathione S-transferase mu 4; MOV10, si:dkeyp-38g6.3 (Moloney leukemia virus 10); NAPA, N-ethylmaleimide sensitive fusion protein attachment protein alpha; PGDS, prostaglandin D2 synthase, hematopoietic; PICK1, hypothetical protein LOC791503; PPME1, zgc:56239 (protein phosphatase methylesterase 1); PSME4, hypothetical LOC561538 (proteasome (prosome, macropain) activator subunit 4); RAB1A, RAB1A member RAS oncogene family; RABL4, RAB, member of RAS oncogene family-like 4; RAP1A, RAP1A, member of RAS oncogene family; SARS2, seryl-tRNA synthetase 2, mitochondrial; SLMO2, slowmo homolog 2 (Drosophila) (similer to kiser); VAPA (VAMP (vesicle-associated membrane protein)-associated protein A, 33 kDa. Organelles, clockwise from top center: mitochondrion, endoplasmic reticulum, Golgi complex, nucleus.

Functional enrichment analysis based on GO terms for biological process identified 11 significantly enriched terms in the differentially-regulated gene list (Table 5). The most significantly enriched term was “proton transport,” under which all but one of seven differentially-regulated genes was down-regulated. Another notable term was “small GTPase-mediated signal transduction.” “Spermatogenesis” was the twelfth term on the list (p = 0.052). Pathway analysis (Pathway Studio) further illustrated the results of the enrichment analysis (Figure 4): most affected transcripts were down-regulated (blue color), many of these transcripts are found in the mitochondria, and there were a large number of associations with the cell processes “respiratory chain,” “cell proliferation,” and “spermatogenesis,” among others.

**Table 5:** Functional enrichment analysis based on Gene Ontology (GO) biological process terms.

Biological Process	GO Term ID	% of DR	% of other	p-value
proton transport	GO:0015992	2.90	0.75	0.005
membrane fusion	GO:0006944	0.83	0.00	0.005
virus induced gene silencing	GO:0009616	0.83	0.00	0.005
receptor clustering	GO:0043113	0.83	0.00	0.005
aromatic compound metabolic process	GO:0006725	1.24	0.13	0.011
seryl-tRNA aminoacylation	GO:0006434	0.83	0.03	0.015
cilium biogenesis	GO:0042384	0.83	0.03	0.015
small GTPase mediated signal transduction	GO:0007264	2.90	1.07	0.023
prostaglandin biosynthetic process	GO:0001516	0.83	0.07	0.029
protein kinase C activation	GO:0007205	0.83	0.07	0.029
neuron differentiation	GO:0030182	0.83	0.10	0.045
spermatogenesis	GO:0007283	1.66	0.52	0.052

“% of DR” refers to the percent of differentially regulated transcripts falling under the term; “% of other” refers to the percent of all other transcripts with GO annotation that fall under the term. P-value is the raw (nominal) p-value from Fisher's exact test.

### Real-time RT-PCR

Efficiencies of the real-time RT-PCR assays here reported ranged from 92.5 percent to 108.6 percent (Table 6), and their correlation coefficients ranged from 0.988 to 0.999. The difference between threshold cycles of the last experimental sample to amplify and the first negative control well to amplify in any reaction was at least 6.49 cycles and 9.83 cycles for –RT and NTC controls, respectively. For each assay, the dissociation curve indicated that a single amplicon was produced. By real-time RT-PCR (n = 4), two of the four genes, Stard7 and TepII, were significantly differentially regulated (p = 0.029 and 0.014, respectively); the direction of regulation was the same as determined by microarray. The fold-change was similar to that determined by microarray for Stard7(1.87 by real-time RT-PCR compared to 2.31 by microarray), but smaller for TepII (5.66 by real-time RT-PCR compared to 29.66 by microarray). For GST, the fold-change was smaller, but the direction of regulation (4.71-fold up-regulated NS) was similar to that determined by microarray (15.24-fold up-regulated NS). This difference in real-time RT-PCR was not significant according to the Kruskall-Wallis test (p = 0.100). For one gene, Ctr1c, the direction of regulation determined by real-time RT-PCR was opposite that determined by microarray, though the difference was essentially zero (1.03-fold down NS by RT-PCR compared to 1.74-fold up NS by microarray). This change was not significant by Kruskall-Wallis (p = 0.443). Therefore, RT-PCR results were similar to microarray, though each transcript's fold change and statistical significance was reduced when measured by RT-PCR, compared to microarray.

**Table 6:** Comparison of microarray and real-time RT-PCR results.

	Microarray			Real-Time RT-PCR			
Gene	Fold Change	Direction	p>F	Fold Change	Direction	p>t	Efficiency
Ctr1c	1.75	up	0.029	1.03	down	0.443	108.2%
TepII	29.66	up	0.020	5.66	up	0.014	92.5%
GST	15.24	up	0.009	4.71	up	0.100	95.1%
Stard7	2.32	down	0.024	1.89	down	0.029	100.7%

Real-time RT-PCR values were normalized to 18S rRNA (18S rRNA efficiency  = 108.6%). “Direction” of regulation is given for nearshore samples, with respect to offshore. Transcripts: Ctr1c, copper transporter 1c; TepII, thiolester containing protein II; GST, Similar to Glutathione S-transferase; Stard7, StAR-related lipid transfer (START) domain containing 7.

### Tissue metal burdens

**Figure 5:**
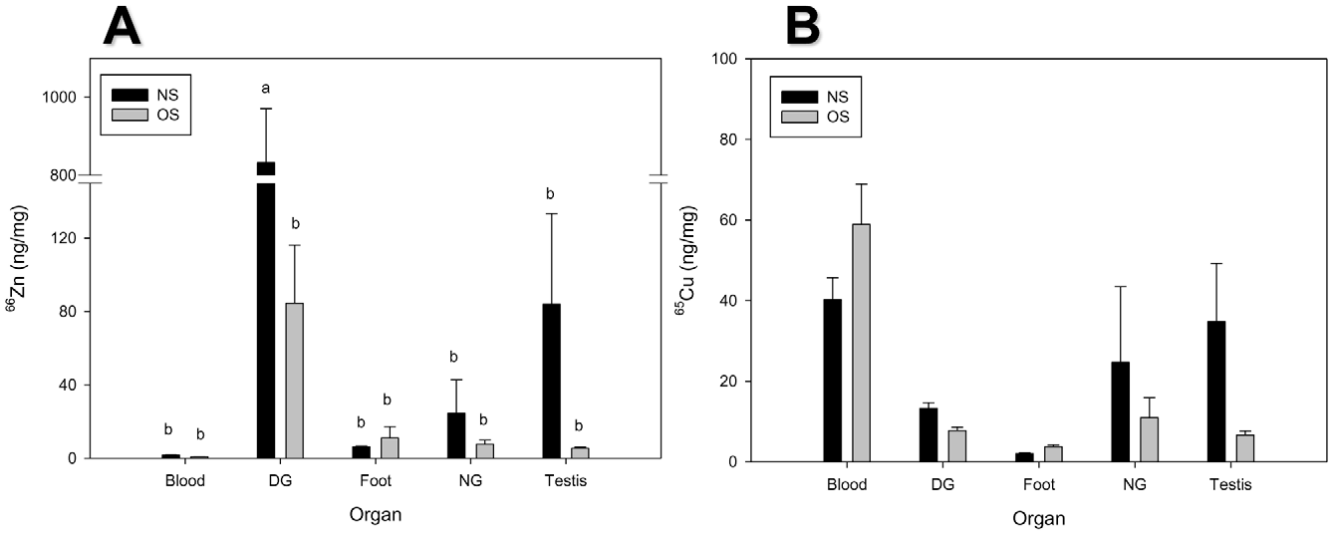
Tissue distribution of Zn (A) and Cu (B) in offshore (OS) and nearshore (NS) conchs. Letters indicate significant difference in 2-way ANOVA, with the two factors tissue and location, followed by Tukey-Kramer HSD (p<0.05). Note different y-axis for Cu and Zn. Break in Zn data (A) omits 150–800 ng/mg. DG  =  digestive gland; NG  =  neural ganglia.

ICP-MS results for all nine analytes are given in Table S3 (sample size varies: n = 2–8/group, specifically enumerated in Table S3). ^66^Zn was present at a significantly higher level in the digestive gland of NS conchs (831.85 ng/mg) than OS conch digestive gland (84.53 ng/mg), or any other tissue at either site (Figure 5A). In addition, although not statistically significant, the concentration of Zn in the NS testis (83.96 ng/mg) was approximately 15-fold higher than in the OS testis (5.43 ng/mg) (Figure 5A). ^65^Cu, conversely, was not significantly higher in any of the NS tissue means compared to the corresponding OS means. However, there was a non-significant (p = 0.65), approximately five-fold difference between ^65^Cu levels in NS (34.77 ng/mg) and OS (6.60 ng/mg) gonad (Figure 5B, Table S3). In the tissue term of the two-way ANOVA, concentrations of ^58^Ni, ^66^Zn, ^111^Cd, and ^238^U were significantly higher in digestive gland than any other tissue. ^118^Sn, despite being detected only at very low concentrations in these samples, was found at its highest concentrations in the neural ganglia. ^65^Cu levels were highest in the blood, which in molluscs contains a copper-based hemocyanin pigment [27].

### Correlations among microarray, histology, and metal data

Correlation analysis was based on testis histological conditions (n = 7–8), metal concentrations in testis and digestive gland (n = 3–8), and expression levels of differentially-regulated transcripts under the GO biological processes spermatogenesis and small GTPase-mediated signal transduction as determined by microarray (n = 3) (Table 7). SI was significantly inversely correlated with digestive gland Zn (ρ = −0.655), and inversely correlated with digestive gland Cu (ρ = −0.509), though this was not statistically significant (p = 0.110). Digestive gland Zn was also significantly and inversely correlated with four of the 11 transcripts included in the analysis; gonad Zn was correlated with two of the 11. SI was significantly correlated with six of the 11 genes in the analysis.

**Table 7:** Non-parametric correlations among Spermatogenic Index, metal concentrations, and transcript expression levels.

	SI	DG Zn	Testis Zn	DG Cu	Testis Cu	PSME4	KISER	DNAJB13	DEGS1	RRAS	RAB1B	RAB1A	TIAM1	RABL4	ARL1	4R79.2
SI																
DG Zn	**−0.655**															
Testis Zn	−0.382	**0.733**														
DG Cu	−0.509	**0.836**	**0.661**													
Testis Cu	−0.345	0.333	−0.164	0.491												
PSME4	0.771	−0.600	−0.800	−0.429	0.100											
KISER	0.771	−0.771	−0.700	−0.543	−0.100	0.714										
DNAJB13	**0.886**	−0.543	−0.600	−0.314	−0.314	**0.943**	0.771									
DEGS1	**0.829**	**−0.829**	−0.600	−0.600	−0.600	0.600	**0.943**	0.714								
RRAS	**0.829**	−0.657	**−0.900**	−0.371	0.200	**0.943**	0.771	0.771	0.657							
RAB1B	**0.886**	**−0.886**	−0.700	−0.600	−0.600	0.600	0.771	0.771	**0.886**	0.714						
RAB1A	0.714	**−0.886**	**−1.000**	−0.657	0.100	0.771	0.771	0.657	0.714	**0.886**	**0.829**					
TIAM1	**−0.943**	0.771	0.600	0.429	0.500	−0.657	**−0.886**	−0.771	**−0.943**	−0.771	**−0.943**	−0.771				
RABL4	**0.829**	**−0.829**	−0.600	−0.600	−0.500	0.600	**0.943**	0.714	**1.000**	0.657	**0.886**	0.714	**−0.943**			
ARL1	0.771	−0.771	−0.700	−0.543	−0.100	0.714	**1.000**	0.771	**0.943**	0.771	0.771	0.771	**−0.886**	**0.943**		
4R79.2	0.657	−0.657	−0.800	−0.600	0.100	**0.943**	0.771	**0.886**	0.657	**0.829**	0.543	0.714	-0.600	0.657	0.771	

“SI” refers to Spermatogenic Index, determined histologically. “DG” refers to digestive gland. Transcripts: PSME4, proteasome activator subunit 4; KISER, similar to kiser; DNAJB13, DnaJ related subfamily B member 13; DEGS1, degenerative spermatocyte homolog 1 lipid desaturase; RRAS, related Ras viral oncogene homolog; RAB1B, Ras-related protein 1B; RAB1A, RAB1A member Ras oncogene family; TIAM1, T-cell lymphoma invasion and metastasis 1; RABL4, Rab member of Ras oncogene family-like 4; ARL1, ADP ribosylation factor-like 1; 4R79.2, 4R79.2 hypothetical protein. Boldface values indicate significance (p<0.05).

## Discussion

The testis histological data gathered in this study show a strong difference between NS and OS conchs in terms of development throughout the reproductive season (Table 3, Figure 2). Queen conchs from NS sites had dramatically less spermatogenic tissue in relation to OS conchs in both February and June. Additionally, spermatogenesis was somewhat reduced in February and markedly reduced in June in NS conchs, during the peak of the conch reproductive season. The SI values give a clear picture of the reduction in spermatogenic capability of NS conchs during both February and June, and highlight the decrease in spermatogenic capability of NS conchs between February and June. Although all NS conchs were undergoing active spermatogenesis in February, there is evidence of developmental delay in February, as a lower percentage of NS conchs were Spawning Capable compared to OS conchs, as well as evidence of significant regression from February to June. The histological data from June collections show that NS conchs are unable to maintain reproductive capability throughout the spawning season. While all OS conchs collected in June had high SI values and were Spawning Capable, only 20% of the NS conchs were Spawning Capable, and all had very low SI values. The current study only presents this histological data as a physiological anchor for gene expression at two sites in the Florida Keys. While we acknowledge that site-specific effects may play a large role in testis development, these observations mirror results from conchs collected at similar NS and OS areas of the Florida Keys in 1999 [2], suggesting that NS conchs show a persistent, long-term reduction in reproductive capability. Moreover, the histology here reported showed a more dramatic reduction than that reported for 1999.

These results complement the results of our microarray and ICP-MS experiments. NS conch testis transcription differed from OS in the GO biological processes proton transport (GO:0015992), spermatogenesis (GO:0007283), small GTPase-mediated signal transduction (GO:0007264), and others (Table 5, Figure 4). This supports specific hypothesis (1), and also suggests that inhibition of small GTPase (Ras)-mediated signaling in NS testis contributes to NS reproductive failure. ICP-MS analysis indicated that Cu and Zn were elevated in some NS conch tissues, providing preliminary support for specific hypothesis (2), and creating the hypothesis that Cu and Zn may be a causative factor in reproductive failure of NS conchs in the Florida Keys. It is important to note that site-specific differences in metal concentrations and gene expression surely exist. Future studies will incorporate metal and gene expression data from additional sites to determine whether differences in these parameters are as consistent as the histological differences observed throughout the NS and OS Florida Keys.

### Conch testis gene expression

The gene expression analysis in the conch testis reveals, logically, that spermatogenesis-associated transcripts are down-regulated NS. Correspondingly, mitochondrial transcripts are significantly down-regulated in NS testes. The effects on proton transport identified by the GO enrichment analysis could be either a cause or a result of the observed reduction in spermatogenesis in NS testes, given the important role of mitochondria in spermatozoa and in sperm maturation [28]–[30]. Our finding is likely the result of the reduction in mature spermatozoa, and consequent numeric reduction in mitochondria, in NS testes as opposed to OS.

Under the Biological Process GO:0007283, spermatogenesis, we identified differentially regulated transcripts with major roles in spermatogenesis in species ranging from *Drosophila* to humans, including degenerative spermatocyte homolog 1 (DEGS1) [31]; Similar to Kiser (homologous to slowmo) [32]; proteasome activator subunit 4 (PSME4/PA200) [33]; DnaJ related, subfamily B, member 13 (DNAJB13) [34], [35], which is also related to the TSARG genes in rats [36] and mice [37]; and nuclear autoantigenic sperm protein (histone-binding) (NASP) [38]. These genes, important for the process of spermatogenesis in a wide range of species, appear to be conserved in queen conch, and were all down-regulated NS in the present study.

A surprising result of the GO enrichment analysis was the enrichment of the term “small GTPase-mediated signal transduction.” Most of the genes under this term are related to Ras-GTPases, proto-oncogenes involved in mammalian tumor formation and developmental disorders [39]. Seven genes that fall under this GO term were differentially regulated in our experiment, including related Ras viral oncogene homolog (Rras); Ras related protein 1b (Rap1b); RAB1A member of Ras oncogene family; T-cell lymphoma invasion and metastasis 1 (TIAM1); RAB member of ras oncogene family 4-like (RABL4); ADP ribosylation factor-like 1 (ARL1); and 4R79.2, a hypothetical GTP-binding protein identified in *Caenorhabditis elegans*. All of these genes are down-regulated with the exception of TIAM1 (Table S2). Ras function has been described in invertebrates including ascidians, for which Ras signaling is involved in embryonic tissue development [40], and *Drosophila*, for which Rap1 is involved in cell adhesion and polarity during epidermal growth factor receptor-mediated tissue growth [41]. Ras genes are also known to be involved in vertebrate and invertebrate testis development. The Ras-cyclin D2 pathway is involved in mouse spermatogonial stem cell development *in vitro* [42]. MAPK and Rap-GEF signaling pathways are also involved in testis development and renewal in *Drosophila* [43]. Therefore, Ras-GTPase signaling may play a major role in conch testis tissue growth and differentiation. Histological SI was correlated with six of the 11 differentially regulated transcripts involved in spermatogenesis or small GTPase-mediated signaling (Table 7). This suggests that transcription of these genes is indicative of the overall maturation of the testis tissue in queen conchs, and that perturbation of normal transcription of these genes is detrimental to spermatogenesis.

Transcripts evaluated by real-time RT-PCR were selected based on their differential regulation between NS and OS, according to the microarray study (Table S2) and their varied and interesting biological functions. GO biological processes of these gene products include: Ctr1c, copper transmembrane transport; TepII, antibacterial humoral response; GST, glutathione metabolic process; Stard7, no biological process (but related to steroidogenic acute regulatory (StAR) protein). The results of our real-time RT-PCR assays were largely successful in validating the changes observed in the microarray study. TepII, GST, and Stard7 were confirmed by real-time RT-PCR, though the GST result was not statistically significant. Ctr1c, however, was essentially unchanged between NS and OS samples in real-time RT-PCR, with a 1.03-fold change in the direction opposite that determined by microarray. The difference in results between platforms is possibly due in part to the small sample size (n = 4) used for both assays; increased sample size would lend power to the analyses. Unfortunately, permitting regulations limit sample size for a protected species such as *S. gigas*. It is also possible that for Ctr1c our probe was designed to a region with homology to other proteins or isoforms in the SLC31 family of copper transporters, causing the lack of consistency between microarray and real-time RT-PCR. Changes in TepII, GST, and Stard7 may indicate that stressors affecting NS conchs cause changes in immune response, xenobiotic metabolism/redox balance, and steroidogenesis, respectively. However, these are single gene changes, and so should be interpreted carefully.

### Potential role of metals as a reproductive stressor

Our ICP-MS data indicated that Cu and Zn, two known reproductive toxicants in gastropods, were elevated in some NS conch tissues. Our study also included other analytes with known toxic effects, including Ni, Ag, Cd, Sn, Hg, and U. Sr was included due to its role in shell-building; it is known to be physiologically beneficial in gastropods at low doses, but toxic at high levels [44]. However, few differences were observed for the latter seven analytes. The effects of Cu and Zn on gastropod reproductive output have been well-documented, although most examples relate to females. In laboratory exposures, Cu has resulted in reduced fecundity in *Helix aspersa* [6], reduced egg-laying and a dose-dependent reduction in hatching in *Pomacea palludosa* [7], and, as copper oxychloride, reduced oocyte number in the ovotestis of *Helix aspersa* [8]. Zn exposures, likewise have impacted reproduction in numerous studies, resulting in reduced fecundity and population growth rate in *Valvata piscinalis* [10], reduced fecundity in *Helix aspersa* [6], and, as an effluent containing Zn, Cd, and Fe, mortality and reduced egg laying in *Lymnaea palustris* [9].

General and point sources of heavy metals in south Florida include storm water runoff, roadway contaminants, septic system leachate, and boats, which may be responsible for high levels of Hg, Pb, Zn, and Cu in waterways [12]. Elevated Cu, Zn, Cr, Hg, Pb, and Ni levels have been identified in Biscayne Bay, adjacent to the city of Miami, as well as at the outflow of canals [11]. Additionally, heavy metals including Cu and Zn have been detected in sediments and seagrass beds, both habitats occupied by conchs, as well as in surface waters at multiple sites throughout south Florida, with Cu sometimes exceeding guidelines for aquatic life and sediment quality [13]. Taken together, this information suggests that potential sources of Cu and Zn contamination exist in the Florida Keys and are likely to be primarily on land or close to shore, further supporting the plausibility of these metals interfering with NS testis development.

In the present study, Zn was elevated in the digestive gland, and possibly in the gonad, of NS conchs (Figure 5A). Coupled with the knowledge that Zn causes reduced fecundity in other gastropod species [6], [9], this finding suggests that Zn may contribute to the reproductive failure of NS conchs. The observed NS digestive gland mean concentration of 831.85 ng Zn/mg tissue is similar to the body burden observed (approx. 200–500 µg Zn/g tissue) in effluent treatments resulting in mortality and reduced fecundity in *Lymnaea palustris* [9]. While available data in the literature focus on female-mediated reproductive inhibition measured as reduced fecundity, studies of fecundity may miss mechanistic effects in both males and females. Further, while the gonad is the apparent site of action for any potential toxicant, accumulation of Zn in the digestive gland in the present study is also likely to be a significant finding. The digestive gland is adjacent to the gonad and is believed to be a site of metal accumulation and detoxification in gastropods [45]–[48]. While a recent study indicates that Zn concentrations in the testis of the Japanese eel *Anguilla japonica* track the progression of spermatogenesis [49], it is important to note that an excess of Zn from external sources could still have a deleterious effect, as is possible in the present study. The relationship between Zn and spermatogenesis is likely complex, and should be the subject of further study.

No significant differences in Cu concentrations within any tissue were found between NS and OS. The mean concentration of 34.77 ng Cu/mg tissue observed in NS conch testis in this study is still only a fraction of the toxic levels accumulated in studies by Rogevich et al. [7] (396.60 ng Cu/mg tissue) and Snyman et al. [8] (260.47 ng Cu/mg tissue), but is approximately five times the OS mean of 6.60 ng Cu/mg tissue. Further, the aforementioned studies measured whole body Cu rather than tissue-specific accumulation. Blood levels of Cu in our study (40.18 ng Cu/mg tissue NS, 58.90 ng Cu/mg tissue OS) were the highest of any tissue, and it would be difficult to separate the Cu contribution of hemocyanin in a tissue to the amount actually bound up in cells. In other words, blood Cu bound in hemocyanin might obscure differences between tissues. Therefore, Cu might still be a factor in NS reproductive failure, and future studies will attempt to test this possibility. It should also be noted that many environmental factors could be considered stressors in a complex environmental mixture, and as with all real-world situations, multiple stressors are likely involved at our NS sites. The inverse correlations between Cu and Zn concentrations in the digestive gland and SI (Table 7) provide support for the argument that accumulation of metals, including Zn and possibly Cu, in the conch digestive gland affects development of the conch testis. These hypotheses will be examined in future studies.

### High-throughput sequencing for gastropod transcriptomics

The approximately 60,000 extant gastropods make up the largest class within the 100,000-member phylum Mollusca, the second-largest animal phylum [50]. However, very little work has been done in the area of gastropod genomics. A PubMed search for “gastropod microarray” on 16 July 2010 yielded only 14 results, one of which was non-germane. Two of the remaining 13 papers discussed toxicogenomics as a tool for understanding endocrine disruption in invertebrates [51], [52]. The remaining 11 papers applied to only five genera of gastropods: *Helix* [53], *Lymnaea* [54], *Haliotis* [55], *Aplysia* [56], and *Biomphalaria* [57], [58], or to schistosomes that use both humans and gastropods as hosts [59]–[63]. A fielded search for “gastropoda[organism]” on GEO yielded only 13 results, consisting of the two submissions here reported, in addition to two platforms (GPL3635 and GPL3636) and two gene expression datasets (GSE4628 and GSE18783) for *Aplysia californica*, one platform (GPL7421) and one gene expression dataset (GSE13039) for *Haliotis asinina*, and two platforms (GPL9129 and GPL9483) and two gene expression datasets (GSE16596, GSE18705, and GSE22037) for *Biomphalaria glabrata*. The use of high-throughput sequencing allowed us to make a significant contribution to this growing field. Still, aside from several heavily studied genera, one of which (*Biomphalaria*) has direct importance for human health, the entire realm of gastropod genomics remains to be developed.

### Conclusions

This study has provided new information regarding the reproductive failure of NS conchs in the Florida Keys. The major findings of this study include the following: first, that failure of NS conchs to reproduce is coupled with a reduction in NS testis development, as previously reported [2], and premature regression of NS testis. Second, the microarray results indicate that reduced testis tissue in NS male conchs is concurrent with a decrease in the expression of many genes related to spermatogenesis and mitochondrial function. Transcription of small GTPase-related signaling genes is clearly affected, and this may contribute to the lack of testis tissue development, but this requires further study. Finally, this study supports the hypothesis that heavy metals may contribute to the reproductive failure of NS conchs. Zn and possibly Cu are elevated in the NS conch digestive gland, and Zn may be elevated in the testis. Given that Zn and Cu are known to reduce gastropod fecundity, the possibility that these same metals may also inhibit gametogenesis in both males and females merits further consideration.

Note that this study characterized effects of the NS environment on reproductive tissue of male conchs. While many gastropod reproduction studies rely on egg-laying (i.e. female-mediated effects) as the measure of average reproductive success [6]–[10], the phenomenon observed in the NS Florida Keys affects both males and females [2], [3]. Conchs rely on mate-pairing and copulation [4], rather than broadcast spawning or other mating strategies that would require fewer reproductive males. Logically, this lack of male reproductive maturity could have a significant impact on the conch population. Future studies will aim to assess transcriptional effects on the ovaries of affected NS females, in addition to males. Although the testicular regression in NS conchs appears to be a persistent problem in the Florida Keys, it is apparently reversible at the level of the individual, as many NS conchs transplanted to OS areas become Spawning Capable [2]. This suggests that transcriptional effects, which can immediately and transiently respond to environmental factors, can play an important role in understanding the disparity in conch reproduction from NS to OS, as well as identifying responsible factors. Therefore, the combination of microarray studies with more traditional approaches will yield useful information for managers as they work to facilitate the recovery of NS queen conch populations in the Florida Keys.

## Supporting Information

Table S1 Validation of 18S rRNA as a reference gene for real-time RT-PCR. “Tukey” denotes whether interaction term tissue*OS/NS is significantly different by ANOVA (only if p<0.05) followed by Tukey-Kramer HSD for multiple comparisons. Within each analyte, values not connected by the same letter are significantly different. *NS samples for 06/2007 were contaminated with digestive gland. Microarray and real-time RT-PCR reported in the present study was conducted with 02/2007 samples.
(0.03 MB DOC)

Table S2 List of all differentially regulated probes from the microarray experiment (p<0.05, FDR = 5%). No gene title is given for probes with insufficient annotation. “Diff of treatment  =  (NS)-(OS)” gives the log2-transformed fold change with respect to NS (OS as control). P-value as determined by one-way ANOVA (FDR = 5%).
(0.15 MB XLS)

Table S3 ICP-MS analysis. “Tukey” denotes whether interaction term tissue*OS/NS is significantly different by ANOVA (only if p<0.05) followed by Tukey-Kramer HSD for multiple comparisons. Within each analyte, values not connected by the same letter are significantly different.
(0.11 MB DOC)
