# Supplementary material for: Queen Conch (Strombus gigas) Testis Regresses during the Reproductive Season at Nearshore Sites in the Florida Keys
Source: PLoS One. 2010 Sep 15;5(9):e12737. doi: 10.1371/journal.pone.0012737 (PMC2939879; doi:10.1371/journal.pone.0012737)
Supplement: Table S1 — Validation of 18S rRNA as a reference gene for real-time RT-PCR. “Tukey” denotes whether interaction term tissue*OS/NS is significantly different by ANOVA (only if p<0.05) followed by Tukey-Kramer HSD for multiple comparisons. Within each analyte, values not connected by the same letter are significantly different. *NS samples for 06/2007 were contaminated with digestive gland. Microarray and real-time RT-PCR reported in the present study was conducted with 02/2007 samples. (0.03 MB DOC) [file pone.0012737.s001.doc]

| Collection | Mean 18S copies/ng (NS) | SEM (NS) | N (NS) | Tukey | Mean 18S copies/ng (OS) | SEM (OS) | N (OS) | Tukey |
| --- | --- | --- | --- | --- | --- | --- | --- | --- |
| 02/2007 | 249250.0 | 48436.2 | 4 | ab | 252000.0 | 26498.4 | 4 | ab |
| 06/2007 | 111882.5 | 55357.8 | 4 | bc* | 344000.0 | 35000.0 | 2 | a |
| 03/2009 | 33624.5 | 17324.5 | 2 | c | 210785.4 | 18636.2 | 7 | abc |
