# Supplementary material for: Queen Conch (Strombus gigas) Testis Regresses during the Reproductive Season at Nearshore Sites in the Florida Keys
Source: PLoS One. 2010 Sep 15;5(9):e12737. doi: 10.1371/journal.pone.0012737 (PMC2939879; doi:10.1371/journal.pone.0012737)
Supplement: Table S3 — ICP-MS analysis. “Tukey” denotes whether interaction term tissue*OS/NS is significantly different by ANOVA (only if p<0.05) followed by Tukey-Kramer HSD for multiple comparisons. Within each analyte, values not connected by the same letter are significantly different. (0.11 MB DOC) [file pone.0012737.s003.doc]

| Analyte | Organ | n (NS) | Mean (NS) (ng/mg) | SEM (NS) | Tukey | n (OS) | Mean (OS) (ng/mg) | SEM (OS) | Tukey |
| --- | --- | --- | --- | --- | --- | --- | --- | --- | --- |
| 58Ni | Blood | 8 | 0.11 | 0.028 |  | 5 | 0.06 | 0.027 |  |
|  | DG | 4 | 24.52 | 7.382 |  | 6 | 30.24 | 4.486 |  |
|  | Foot | 5 | 0.45 | 0.425 |  | 6 | 0.15 | 0.103 |  |
|  | NG | 7 | 2.47 | 2.075 |  | 6 | 0.53 | 0.120 |  |
|  | Testis | 6 | 3.24 | 1.386 |  | 5 | 1.41 | 0.248 |  |
| 65Cu | Blood | 8 | 40.18 | 5.514 |  | 5 | 58.90 | 10.008 |  |
|  | DG | 4 | 13.28 | 1.395 |  | 6 | 7.72 | 0.874 |  |
|  | Foot | 5 | 2.00 | 0.264 |  | 6 | 3.72 | 0.468 |  |
|  | NG | 7 | 24.70 | 18.745 |  | 6 | 10.97 | 4.919 |  |
|  | Testis | 6 | 34.77 | 14.431 |  | 5 | 6.60 | 1.064 |  |
| 66Zn | Blood | 8 | 1.66 | 0.304 | b | 5 | 0.80 | 0.101 | b |
|  | DG | 4 | 831.85 | 138.771 | a | 6 | 84.53 | 31.689 | b |
|  | Foot | 5 | 6.17 | 0.450 | b | 6 | 11.05 | 6.250 | b |
|  | NG | 7 | 24.42 | 18.350 | b | 6 | 7.69 | 2.168 | b |
|  | Testis | 6 | 83.96 | 49.359 | b | 5 | 5.43 | 0.698 | b |
| 88Sr | Blood | 8 | 7.44 | 0.548 |  | 5 | 7.18 | 0.395 |  |
|  | DG | 4 | 21.55 | 5.557 |  | 6 | 20.61 | 1.695 |  |
|  | Foot | 5 | 8.71 | 0.632 |  | 6 | 7.59 | 1.260 |  |
|  | NG | 7 | 23.99 | 15.192 |  | 6 | 8.75 | 3.578 |  |
|  | Testis | 6 | 18.34 | 9.785 |  | 5 | 11.91 | 2.284 |  |
| 107Ag | Blood | 8 | 0.08 | 0.019 | b | 5 | 0.80 | 0.091 | b |
|  | DG | 4 | 0.04 | 0.010 | b | 6 | 3.90 | 1.585 | a |
|  | Foot | 5 | 0.04 | 0.012 | b | 6 | 0.14 | 0.063 | ab |
|  | NG | 7 | 0.72 | 0.639 | b | 6 | 0.21 | 0.115 | b |
|  | Testis | 6 | 0.18 | 0.038 | b | 5 | 0.26 | 0.053 | b |
| 111Cd | Blood | 8 | 0.05 | 0.011 | c | 5 | 0.03 | 0.010 | c |
|  | DG | 4 | 4.76 | 0.746 | b | 6 | 9.58 | 1.084 | a |
|  | Foot | 5 | 0.44 | 0.053 | c | 6 | 0.51 | 0.039 | c |
|  | NG | 7 | 0.98 | 0.739 | c | 6 | 0.30 | 0.050 | c |
|  | Testis | 6 | 0.67 | 0.300 | c | 5 | 1.35 | 0.939 | c |
| 118Sn | Blood | 8 | 0.01 | 0.000 |  | 5 | 0.01 | 0.000 |  |
|  | DG | 4 | 0.02 | 0.004 |  | 6 | 0.03 | 0.010 |  |
|  | Foot | 5 | 0.02 | 0.001 |  | 6 | 0.02 | 0.003 |  |
|  | NG | 7 | 0.12 | 0.044 |  | 6 | 0.09 | 0.027 |  |
|  | Testis | 6 | 0.04 | 0.009 |  | 5 | 0.02 | 0.005 |  |
| 202Hg | Blood | 8 | 0.02 | 0.003 | b | 5 | 0.02 | 0.005 | ab |
|  | DG | 4 | 0.03 | 0.016 | ab | 6 | 0.28 | 0.074 | ab |
|  | Foot | 5 | 0.43 | 0.166 | a | 2 | 0.05 | 0.024 | ab |
|  | NG | 7 | 0.25 | 0.124 | ab | 6 | 0.18 | 0.079 | ab |
|  | Testis | 6 | 0.05 | 0.008 | ab | 5 | 0.25 | 0.085 | ab |
| 238U | Blood | 8 | 0.03 | 0.006 |  | 5 | 0.01 | 0.000 |  |
|  | DG | 4 | 2.03 | 0.535 |  | 6 | 1.10 | 0.247 |  |
|  | Foot | 5 | 0.02 | 0.001 |  | 6 | 0.02 | 0.003 |  |
|  | NG | 7 | 0.23 | 0.151 |  | 6 | 0.12 | 0.035 |  |
|  | Testis | 6 | 0.32 | 0.172 |  | 5 | 0.03 | 0.005 |  |
